# Comanche County Medical Society

**Published:** 1892-07

**Authors:** J. D. Wingate

**Affiliations:** Secretary


### COMANCHE COUNTY MEDICAL SOCIETY.

The following named physicians met in the office of Dr. C. F. Paine for the purpose of organizing a County Medical Society: C. F. Paine, C. F. Rogers, W. F. James, J. F. McCarty, J. J. Eargle and J. D. Wingate. The purpose of the organization is to discuss among themselves the ways and means, unexhausted and inexhaustible, for the alleviation of suffering, and the prevention of disease; and, second, their purpose is, by harmonious action and ethical bearing, to show to the public that we propose as a profession to keep abreast with the times, and improve the opportunity offered us.

Election of officers resulted in Dr. C. F. Paine, President; J. J. Eargle, Vice-President, and J. D. Wingate, Secretary and Treasurer.

Roll of membership was then opened with the following names thereto attached: C. F. Paine, J. J. Eargle, C. F. Rogers, J. T. Eargle, W. F. James, J. F. McCarty, R. A. Miller, Dublin, —— Fowler, Fleming, J. D. Wingate.

On motion of Dr. C. F. Rogers, the President appointed Drs. Eargle and Wingate to prepare papers to be read before the society at its next regular meeting.

*Resolved*, That a synopsis of the minutes of this meeting be furnished by the Secretary to DANIEL'S TEXAS MEDICAL JOURNAL for publication; and further, that an earnest invitation be hereby published to all physicians in Comanche and adjoining counties to be present and join us at our next regular meeting, to be held in Comanche Tuesday, August 2, at 2 oclock p. m. This being the time for the adoption of our constitution and by-laws, we desire all the physicians who can to be present and help in this important step.

J. D. WINGATE,
Secretary.

### THE MISSISSIPPI VALLEY MEDICAL ASSOCIATION.

The Mississippi Valley Medical Association will hold its eighteenth annual session at Cincinnati, Wednesday, Thursday and Friday, October 12th, 13th and 14th, 1892. An excellent program, containing the best names in the valley, and covering the entire field of medicine, will be presented. An address on surgery will be delivered by Dr. Hunter McGuire, of Richmond,

Va., President of the American Medical Association. An address on medicine will be made by Dr. Hobart Amory Hare, Professor of Therapeutics and Clinical Medicine, Jefferson Medical College, Philadelphia. The social as well as the scientific part of the meeting will be of the highest order.

The Mississippi Valley Medical Association possesses one great advantage over similar bodies, in that its organic law is such that nothing can be discussed during the sessions save and except science. All ethical matters are referred, together with all extraordinary business, to appropriate committees—their decisions are final and are accepted without discussion. The constitution and by-laws are comprehensive, and at the same time simple. Precious time is not allowed the demagogue or the medical legislator. The officers of the Pan-American Medical Congress will hold a conference at the same time and place.

CHARLES A. L. REED, M. D., Cincinnati, President.

E. S. MCKEE, M. D., Cincinnati, Secretary.

